# Challenges of small hospitals in Japan during the COVID-19 pandemic

**DOI:** 10.1017/ash.2026.10368

**Published:** 2026-04-20

**Authors:** Chikako Agata, Dang-An Do, Tomoko Yazawa, Yoshiaki Gu

**Affiliations:** 1 Department of Infectious Diseases, Institute of Science Tokyo, Tokyo, Japan; 2 Department of Infectious Disease Emergency Preparedness, Institute of Science Tokyo, Tokyo, Japan; 3 Center for Infectious Disease Education and Analysis (TCIDEA), Institute of Science Tokyo, Tokyo, Japan

## Abstract

An analysis of 630 hospitals and 488 COVID-19 clusters in Tokyo (2020/07-2022/05) found contrasting associations between hospital size and cluster frequency. Compared to larger hospitals (≥600 beds), smaller ones (20-99 beds) reported fewer clusters per facility (0.5 vs 2.0), but a higher cluster number per 1000 beds (6.0 vs 2.5).

The Coronavirus disease 2019 (COVID-19) pandemic has uncovered major weaknesses in the health system worldwide, including financial strain, limited hospital capacity, and staffing shortages.^1^ In Japan, these challenges were intensified by a fragmented and predominantly private healthcare delivery system. Small hospitals with less than 200 beds—representing 93% of private hospitals and 70% of total facilities in Japan—are most vulnerable to the economic impacts of COVID-19.^2^ Early in the pandemic, media reports and cross-sectional surveys highlighted nosocomial transmission as a rapidly emerging threat, frequently forcing facilities to suspend outpatient and emergency services, particularly in small hospitals.^3^ However, few studies have examined the outbreak burden in small hospitals relative to larger hospitals.^2–5^ This study therefore assessed the association between hospital size and the occurrence of COVID-19 clusters in Tokyo, Japan.

We conducted a retrospective cross-sectional study using publicly available data on COVID-19 clusters reported by the Bureau of Social Welfare and Public Health, Tokyo Metropolitan Government, from July 1, 2020 to May 31, 2022. A cluster was defined as five or more confirmed cases—including both patients and healthcare workers—with epidemiological transmission links occurring within the same facility, based on backwards contact tracing guidance from Ministry of Health, Labour and Welfare.^1,2^ The month and year of occurrence were the date the cluster was officially confirmed by the local public health center. Prolonged outbreaks or multiple introductions within the same hospital were categorized as single or multiple clusters based on distinct registrations and epidemiologic linkages determined by both the hospital and public health authorities.

The data set included cluster identification numbers, month and year of occurrence, location (ward or city) of reporting health centers, and numbers of beds in each cluster occurring hospital. When a cluster was identified, hospitals were required to report to the local public health center, which then verified and subsequently submitted the information to the Tokyo Metropolitan Government. The cluster data were reviewed and updated monthly. Hospital size was determined by the number of legally licensed beds registered with regional health authorities in the corresponding Japanese fiscal year (from April 1^st^ to March 31^st^) of the reported clusters. Hospitals were divided into seven categories based on the hospital size (measured by number of licensed bed): 20–99, 100–199, 200–299, 300–399, 400–499, 500–599, and ≥600 beds.

***Two indicators*** were calculated: (i) the ratio of clusters to hospital and (ii) the ratio of clusters to1000 hospital beds, in which the denominators were referred to all 630 hospitals in Tokyo in 2021 and the numerators were drawn from 488 COVID-19 clusters reported during the studied period. Linear regression models with 95% confidence interval (CI) were applied to examine associations between hospital size and each ratio. Analyses were performed using STATA (version 19.5) with statistical significance of *P* < .05. This study was approved by the Institutional Review Board of the Institute of Science Tokyo (Reference: C2022-040, January 17^th^, 2023).

In the results, Figure 1 on cluster numbers in seven categories by year-month shows that clusters occurred in all hospital sizes, but concentrated in small hospitals (20–199 beds) in all epidemic waves. Larger hospitals also experienced clusters, their frequency remained lower and more stable. Regarding associations between hospital sizes and cluster numbers per hospital (left side) and per 1,000 hospital beds (right side), Figure 2 shows that facilities with 20–99 beds reported 0.5 clusters per hospital, while hospitals ≥600 beds experienced a quadrupled number (2.0 clusters per hospital). Linear regression indicates a significant positive correlation between hospital size and the ratio of clusters to hospital (coefficient = 0.30, 95% CI: 0.19 to 0.42, *P* = .001). Conversely, the ratio of clusters to 1,000 beds showed the opposite pattern: hospitals with 20–99 beds reported higher ratios (6.0 vs 2.5) than hospitals ≥600 beds (coefficient = −0.49, 95% CI: −0.87 to −0.12, *P* = .020).

**Figure 1.**
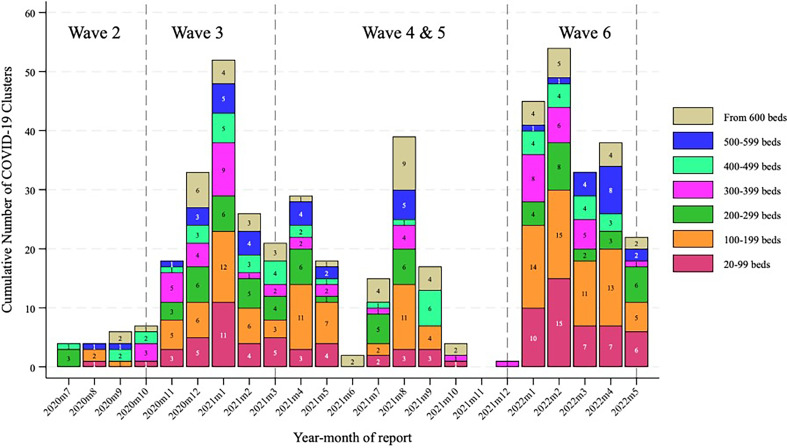
Number of COVID-19 clusters by year-month in Tokyo from July 2020 to May 2022.

**Figure 2.**
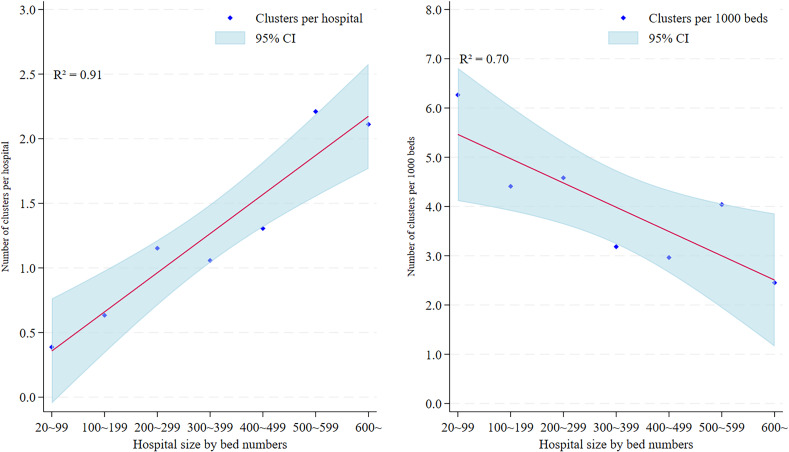
Number of COVID-19 clusters per hospital (left) and per 1,000 hospital beds (right) by size of all hospitals in Tokyo.

These opposite trends reflected two simultaneous challenges faced by small hospitals in Japan. The ***first challenge*** is their greater vulnerability to COVID-19 pandemic despite having limited experience in pandemic response. The higher cluster numbers per bed in small hospitals reflect the disproportionate burden on limited infection prevention and control (IPC) resources, including both infrastructure and workforce capacity. These findings were similar to studies in United States and Brazil, which small hospitals faced prolonged high-occupancy periods, inadequate staffing, insufficient personal protective equipment, and suboptimal ventilation or isolation capacity.^6,7^ Smaller hospitals in Japan also experienced fewer clusters per facility, meaning they had limited opportunities to gain the practical experience in managing COVID-19 cases, which widened the gap in pandemic preparedness compared to larger hospitals.^2^

The ***second challenge*** comes with an inequitable distribution of IPC frameworks between small and large hospitals, which likely reduced the overall efficiency of IPC implementation. A nationwide survey in 2020 on Japan’s reimbursement system for antimicrobial stewardship programs before the COVID-19 found that most IPC financial incentives concentrated in medium- and large-sized hospitals.^8^ Hirao (2020) reported that approximately 60% of infectious disease specialists and certified IPC nurses worked in large hospitals, while smaller hospitals accounted for only 0.4% of such specialists.^9^ A prepandemic study by Nomoto *et al*. (2022) showed that small hospitals achieved significant lower of all domains in the WHO Infection Prevention and Control Assessment Framework compared with medium and large facilities.^10^ This evidence indicates that small hospitals entered the pandemic with substantially weaker IPC capacity and lack of sufficient preparedness. Although national medical expenses in Japan decreased by 2.76% in 2020 compared to previous years—primarily impacting large public hospitals designated for COVID-19 care—the specific consequences for smaller private hospitals remain unclear.^5^ Our results suggests that financial contractions may have further strained IPC infrastructure and staffing in small hospitals, contributing to their high cluster density.

This study is among the few ones to examine the association between hospital size and COVID-19 outbreaks in Japan and worldwide. However, several limitations should be considered. Because data were limited to hospitals reported COVID-19 clusters, causal inference between resource constraints and outbreak occurrences could not be established. Some potential information and selection biases, such as incomplete reporting from hospitals or public health centers, may also have underestimated cluster numbers, particularly in smaller hospitals. Besides, our analysis relied on aggregated facility-level reports rather than detailed individual-level records for patients and staff, we were unable to assess the specific impacts of systemic and clinical factors like workforce depletion, viral strains, temporal shifts in vaccination coverage, or wave-specific public health interventions on the total clusters. Future research should expand the facility sample, incorporate more epidemiological data, and evaluate specific IPC interventions, such as ventilation upgrades, staff training, or mandatory screening programs.

In conclusion, our study identified distinct challenges faced by small hospitals in Tokyo, Japan during the COVID-19 pandemic. While smaller hospitals experienced fewer clusters per facility, they had a higher risk of clusters relative to bed capacity, which reflected vulnerabilities linked to limited IPC infrastructure, workforce shortages, and low participation in financial incentive programs. Larger hospitals, though better equipped, struggled with cluster control strain from high patient volumes and case severity. These findings underscore the need for tailored strategies to strengthen IPC capacity in small hospitals and for systemwide coordination to enhance outbreak preparedness and resilience against future pandemics.

## Supporting information

10.1017/ash.2026.10368.sm001 Agata et al. supplementary material Agata et al. supplementary material

## Supplementary material

The supplementary material for this article can be found at https://doi.org/10.1017/ash.2026.10368.
