# Supplementary material for: Challenges of small hospitals in Japan during the COVID-19 pandemic
Source: Antimicrob Steward Healthc Epidemiol. 2026 Apr 20;6(1):e115. doi: 10.1017/ash.2026.10368 (PMC13104564; doi:10.1017/ash.2026.10368)
Supplement: Agata et al. supplementary material [file S2732494X26103684sup001.pdf]

SUPPLEMENTARY TABLES

Supplementary Table 1. Number of COVID-19 clusters and number of hospitals and its beds in Tokyo

Hospital categories (by bed numbers)	Total hospitals	Total beds	COVID-19 clusters	Clusters per hospital	Clusters per 1000 beds
	(a)	(b)	(c)	c/a	(c/b) * 1000
20~99 beds	235	14523	91	0.4	6.3
100~199 beds	202	29026	128	0.6	4.4
200~299 beds	59	14841	68	1.2	4.6
300~399 beds	52	17276	55	1.1	3.2
400~499 beds	36	15859	47	1.3	3.0
500~599 beds	19	10399	42	2.2	4.0
600+ beds	27	23242	57	2.1	2.5
Total	630	125166	488	0.8	3.9

Supplementary Table 2. Association between the number of clusters per bed/per hospital and the size of hospitals grouped by number of beds

**Dependent Variable**	**Coefficient**	**Std. Error**	**t-value**	**p-value**	**95% Confidence Interval**	**R^2 (Adjusted R^2)**
Clusters per 1000 beds in all hospitals in Tokyo	-0.49	0.15	-3.38	0.020	[-0.87, -0.12]	0.70 (0.63)
Clusters per hospital in all hospitals in Tokyo	0.30	0.04	6.91	0.001	[0.19, 0.42]	0.91 (0.89)

Notes: *Significance level:* $p < 0.05$ *is statistically significant.*
